# 

**Published:** 1908-05-23

**Authors:** 


					? ? p A J ? \ r
*?wirL?4e;j(i fiERAL-:>JWik I MODERN
Telephone Number
Oapedale. London II .MED!CAL JOURNAL. " Gerrard.
N?w SERIES. No. 62, Vol. III. o.TrroIUV
s=r-?j[5)^1134.1 Vol. XLIV. SATURDAY,,
May 23rd, 1908.
PRICE THREEPENCE.

				

